# Supplementary material for: Three-dimensional reconstruction of a whole insect reveals its phloem sap-sucking mechanism at nano-resolution
Source: eLife. 2021 Feb 23;10:e62875. doi: 10.7554/eLife.62875 (PMC8016479; doi:10.7554/eLife.62875)
Supplement: Supplementary file 1. [file elife-62875-supp1.pdf]

Interactive 3D PDF

for

Three-dimensional reconstruction of a whole insect reveals its phloem sap-sucking mechanism at nano-resolution

Xin-Qiu Wang#, Jian-Sheng Guo#, Dan-Ting Li, Yang Yu, Jaco Hagoort, Bernard Moussian, Chuan-Xi Zhang*

Instructions on the use of this interactive 3D PDF

3D interaction is available on MS Windows or Mac OS systems, using Adobe Acrobat or Adobe Acrobat Reader.

★Click on the object to activate the interaction with the object.
★Click on the view buttons in the left panel to display preset views.
★Click on the structures in the 3D model to show the names.
★Click on the buttons in the lower panel to show, hide or make selected structures transparent.

- ■ show
- ■ transparent
- ■ hide

Interaction tools in 3D toolbar enable rotation and resizing of the 3D object.

★Rotate: Hold left mouse button and move mouse.
★Zoom: Scroll mouse wheel.
★Pan: Hold left and right mouse buttons and move mouse.

Internal structure of the brown planthopper, *Nilaparvata lugens*

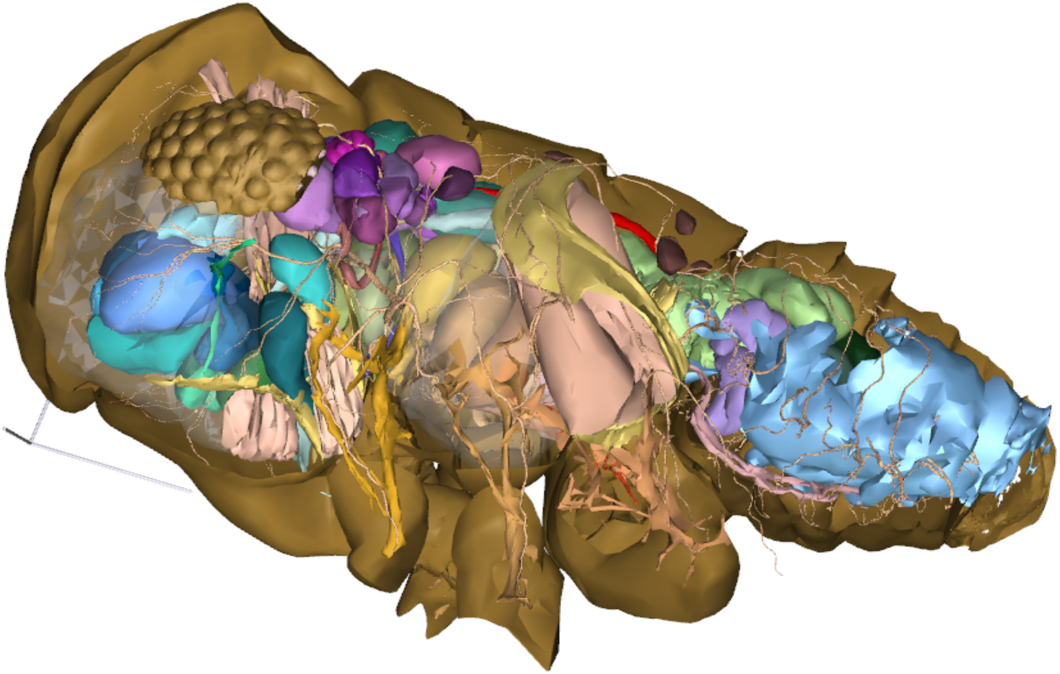

- Lateral view
- Central nervous system
- Cephalic endoskeleton and muscles
- Mouthpart
- Alimentary canal
- Salivary gland
- Tracheal system
- Symbionts
- Four-way tracheal ring

**click on the object to active it*

Selected structure:

cortex
